# Evolution of extrema features reveals optimal stimuli for biological state transitions

**DOI:** 10.1038/s41598-018-21761-8

**Published:** 2018-02-21

**Authors:** Joshua Chang, David Paydarfar

**Affiliations:** 1 Department of Neurology, University of Massachusetts Medical School, Worcester, Massachusetts 01604 USA; 2 Department of Neurology, Dell Medical School, The University of Texas at Austin, Austin, Texas 78701 USA; 3 The Institute for Computational Engineering and Sciences, The University of Texas at Austin, Austin, Texas 78701 USA

## Abstract

The ability to define the unique features of an input stimulus needed to control switch-like behavior in biological systems is an important problem in computational biology and medicine. We show in this study how highly complex and intractable optimization problems can be simplified by restricting the search to the signal’s extrema as key feature points, and evolving the extrema features towards optimal solutions that closely match solutions derived from gradient-based methods. Our results suggest a model-independent approach for solving a class of optimization problems related to controlling switch-like state transitions.

## Introduction

Switch-like transitions play an important regulatory role in biological systems. Cell division, differentiation and apoptosis are controlled by complex molecular signaling networks that can abruptly switch from one pathway to another^1–4^. Electrical signaling of multicellular networks switch between distinct electrical states – quiescence, repetitive firing, and bursting patterns – that are essential for controlling normal movement^5,6^, autonomic function^7^, and behavioral states^8^ or perhaps triggering pathological states like migraines^9,10^. Therapeutic interventions work to exploit switch-like shifts from one state to another, for example, inducing immunity through vaccination with small doses of sub-pathologic viruses^11^, or applying electrical stimuli to defibrillate the heart^12–14^, suppress parkinsonian tremors^15–17^, and abort epileptic seizures^18,19^. Normal and pathological switching are typically governed by highly nonlinear mechanisms, with small input signals inducing large and rapid system responses.

**Figure 1:**
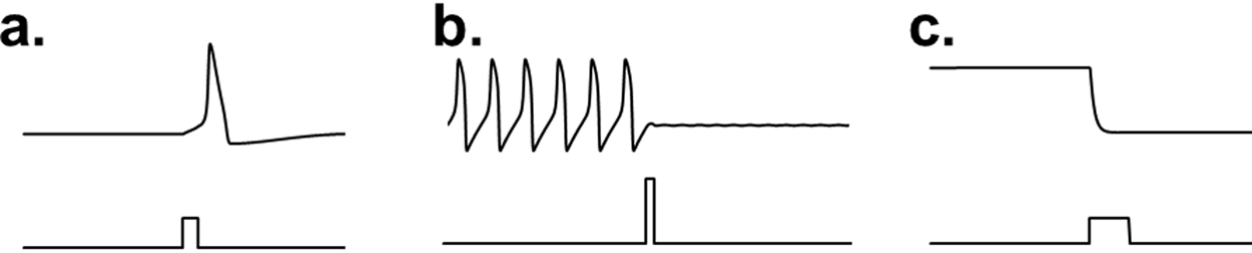
Models illustrating different types of transitions. Panel a shows the Hodgkin-Huxley model transitioning between a stable quiescent state and an unstable active state (action potential), induced by a brief depolarizing pulse. Panel b shows the FitzHugh-Nagumo model transitioning between a stable oscillatory state (repetitive firing) and a quiescent state (with damped subthreshold oscillations), induced by a brief perturbation. Panel c shows the genetic toggle switch model transitioning between two stable steady states of protein production, induced by a pulse of isopropyl β-D-1-thiogalactopyranoside (IPTG). For an explanation of the models, see Supplementary Text S1.

This idea that tiny perturbations can induce large state changes has been the focus of a growing interest in critical transitions^10,20–23^. Some have examined these critical transitions in the context of moving along parameter continua (e.g. transitioning from a monostable system to a bistable system)^22^, while others have examined transitions in a single system from one state to another state^20^. When examining transitions from one state to another, there are a number of different forms, as seen in Fig. 1. Some perturbations induce a specific response trajectory (e.g. an action potential), while others cause a transition from one stable state to another stable state (e.g. limit cycle or fixed point). In all of these cases, an abrupt transition is occurring in the state space caused by a small input stimulus.

A major challenge to understanding these transitions is recognizing what are the key stimulus waveform features that trigger them. While many different stimulus waveforms might push a system across a tipping point to cause a transition in state, they are not all optimal. Defining optimality is highly specific to the system of interest. For electrical stimulation of tissue, clinicians may wish to minimize the peak stimulus intensity to avoid causing tissue damage or energy leakage into neighboring regions, producing detrimental side effects. Other applications may instead seek to minimize total energy consumption, to maximize battery life for implantable devices. In vaccines or chemotherapy, one may seek to minimize the total injected dose to prevent toxicity. In general, therapeutic optimality seeks to achieve a beneficial health outcome while minimizing adverse effects.

Analytical methods for deriving optimal control signals traditionally use calculus of variations^24–26^; broad application of these techniques to biology is limited because validated mathematical models are available in only a few systems, and variational solutions to complex models can be elusive^27,28^. To deal with complex models, researchers have used phase reduction models^28–32^, parameterized stimuli^33^, or simpler conceptual models^24,25^. The majority of biological systems however, do not have models readily available, and as such empirical approaches have explored the system’s responses to simple stimulus waveforms (e.g., rectangular, sinusoidal)^34–37^. The stimulus parameters (e.g., pulse width, frequency) are tuned to achieve the optimal parameters for achieving an outcome. Unfortunately, this approach is also limited because it requires certain assumptions about the optimal shape, leading to a biased search, potentially missing more optimal solutions.

There has been interest in developing unbiased methods to search for optimal stimuli that do not require *a priori* knowledge of the underlying mechanisms or mathematical equations defining the system^38–40^. Since biological control systems are often highly nonlinear and respond to fluctuations over broad ranges of intensity, duration, and timing, finding the optimal control signal can be challenging. One approach is to encode the stimulus as a vector, representing stimulus amplitude over time^39^. The number of dimensions a stimulus requires is calculated by multiplying the duration of the stimulus by the resolution of the stimulus generator. For example, the solution space for a 1-second stimulus with 1-dimensional amplitude generated at a resolution of 1-millisecond has a dimension of 1000. Such high dimensionality of the solution space can rapidly exceed the limits of computational tractability, an example of the “curse of dimensionality”^41,42^.

Here we present a novel evolutionary method for estimating optimal stimuli based on the hypothesis that the optimization problem is vastly simplified by restricting the search algorithm to the signal extrema as key feature points. The algorithm is initially seeded by random signals whose extrema are iteratively perturbed and pruned, thereby sculpting the stimulus waveform towards an optimal shape. We find that this process allows for many degrees of freedom in the early stages, but as the algorithm progresses and extraneous extrema are removed, the degrees of freedom decrease, increasing the probability of finding more optimal solutions. Multiple, independent stochastically-generated initial seeds enable the algorithm to search for both local and global optima. By comparing to numerical solutions from analytically-derived optimal approaches, we demonstrate and validate the algorithm in three models representing different types of transitions (Fig. 1): from quiescence to a single action potential in the Hodgkin-Huxley model^43^; between sustained oscillation and quiescence in the FitzHugh-Nagumo model^44,45^; and between two quiescent states in a genetic toggle switch model^46^. We also apply the algorithm to a more complex problem of finding an optimal stimulus that desynchronizes a group of coupled Hodgkin-Huxley neuronal oscillators, which is not readily solved by traditional approaches.

## Methods

### Stochastic searching with extrema features

This algorithm is based on the hypothesis that optimization of control signals can be aided by restricting the search space to the signal extrema as key feature points, rather than allowing every point of the stimulus to be a unique dimension. The use of extrema in simplifying data has been introduced in other problems in signal processing. In the field of pattern recognition, certain algorithms use the coordinates of robust extrema to decrease the number of points to match^47^. In computer vision, extrema are used as key feature markers for object recognition and multi-scale representation of the original images^48,49^. In many of these algorithms, the extrema are used to highlight key feature points, and thus construct a simplified representation of the original signal retaining only the most important components. This makes the manipulation and usage of the signals more efficient and simpler. We hypothesize that this concept of using extrema to simplify signals is applicable to stochastic search algorithms.

The steps of our algorithm are as follows:

1. Let *µ*_0_ be our initial stimulus, where *µ* = *(µ[1]*, *µ[2]*, *…*, *µ[M])*, *M* being the product of the number of samples per unit time and the duration of the stimulus. This vector is initially randomly generated using a uniform random number generator at each time point.
2. Define the function, *f*, to represent the system in question. Thus, *f (µ)* is the response of the stimulus to a given stimulus.
3. Define *T* to be the set of times *{T*_*1*_, *T*_*2*_, *…*, *T*_*N*_} at which extrema occur in the stimulus. We define an extremum to be an element such that at time *T*_*n*_:
  1 $${\mu }[{T}_{n}-{1}] < {\mu }[{T}_{n}]\,{\rm{and}}\,{\mu }[{T}_{n}+{1}] < {\mu }[{T}_{n}]\,{\rm{or}}\,{\mu }[{T}_{n}-{1}] > {\mu }[{T}_{n}]\,{\rm{and}}\,{\mu }[{T}_{n}+{1}] > {\mu }[{T}_{n}].$$
4. Create a set of intervals, *I*, to be defined as *T*_*n*_ *−* *T*_*n* − *1*_ for *n* = 2, *…*, *N*. Multiply each of these intervals by a random number generated from a Gaussian distribution:
  2 $${I}_{temp,n}={I}_{n}\ast {\mathscr{N}}(1,\,{\sigma }_{I}).$$
5. Rescale the new time intervals such that the total duration matches the original duration:
  3 $${I}_{n}\mbox{'}={I}_{temp,n}\ast {\Sigma }I/{\Sigma }{I}_{temp}.$$
6. Use these new intervals to redefine a new set of times, $T^{\prime}$.
7. Perturb the amplitude of each of the extrema by adding to each peak a randomly generated number:
  4 $${E}_{n}\mbox{'}={E}_{n}\ast {\mathscr{N}}(0,\,{\sigma }_{E})$$
8. Linearly transform all the data points between the original extrema using the new perturbed extrema endpoints to generate a “neighboring stimulus” of the starting seed. Mathematically, a point *(t*, *µ (t))* between the two old extrema *(T*_*n*_, *µ (T*_*n*_*))* and *(T*_*n* + *1*_, *µ (T*_*n* + *1*_*))*, transforms to
  5 $$((\frac{{T}_{n+1}\text{'}-{T}_{n}\text{'}}{{T}_{n+1}-{T}_{n}})\ast ((t-{T}_{n})+{T}_{n}\text{'}),(\frac{\mu \text{'}({T}_{n+1}\text{'})-\mu \text{'}({T}_{n}\text{'})}{\mu ({T}_{n+1})-\mu ({T}_{n})})\ast ((\mu (t)-\mu ({T}_{n}))+\mu \text{'}({T}_{n}\text{'})))$$
  where *(T*_*n*_*’*, *µ’(T*_*n*_*’))* and *(T*_*n* + *1*_*’*, *µ’(T*_*n* + *1*_*’))* are the new perturbed extrema.
9. Apply this new stimulus to the system and measure its system response.
10. Calculate the performance metric (e.g. L^2^-norm, *J* = ∫*µ*^*2*^*dt*) of the neighboring stimulus, penalizing the performance metric if the stimulus does not successfully cause a state transition. For example, we used L^2^-norm:
  6 $$J=\int {{\mu }}^{2}dt+penalty$$
11. Repeat steps 3–7 10 times to produce 10 neighboring solutions based on the starting stimulus.
12. Compare the original starting stimulus with the neighboring stimulus to find the solution with the best performance.
13. Repeat steps 2–11 for a predetermined number of iterations, *L*, using the best performing stimulus found from the previous iteration as the new starting seed in step 2.
14. After *L* iterations, output the stimulus with the best performance metric as the optimal stimulus.

**Figure 2:**
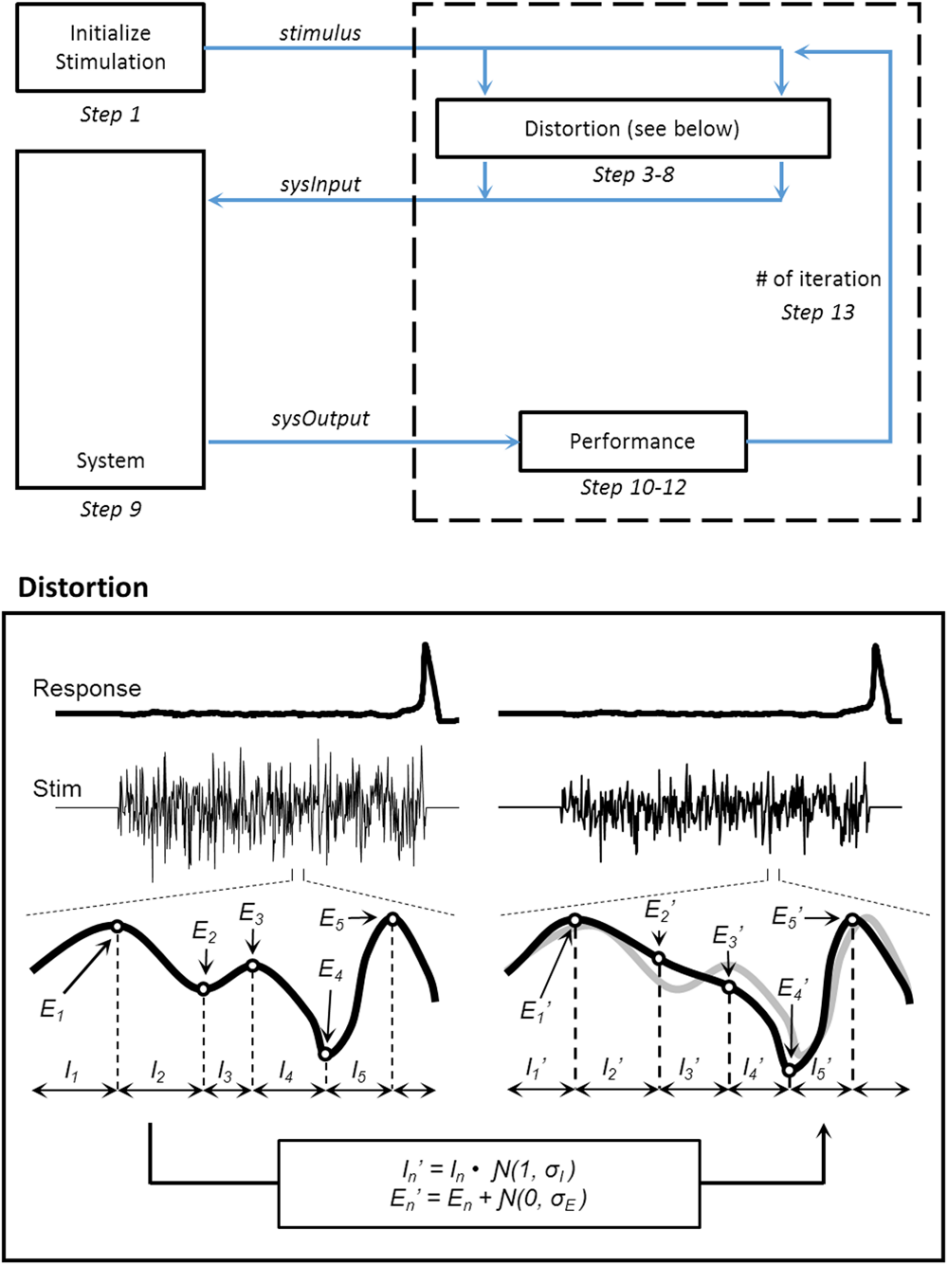
Schematic of the extrema featured stochastic search algorithm (top) along with a detailed representation of the distortion component (bottom). The distortion inset showcases both extrema interval and amplitude distortions before (bottom left) and after (bottom right) a single iteration. *E*_*n*_ and *E*_*n*_^’^ represent the n^th^ extrema of the stimulus before and after the iteration, respectively, and *I*_*n*_ and *I*_*n*_^’^ represent the n^th^ intervals between the respective extrema.

Figure 2 illustrates one iteration of the signal perturbation carried out between Steps 3–6 in a search for the optimal stimulus waveform to trigger an action potential in the Hodgkin-Huxley model. For the uniform random number generator at Step 1, the uniform random number generator should be set such that the noise given to the system just barely causes a switch in states. If an excessive amount of noise is given, state switches may be more easily obtained, but there is a lot more energy to reduce in the iterative process. We used a Gaussian distribution to randomly shift the extrema amplitudes and inter-extrema intervals. The variance of this distribution, the number of neighboring stimuli generated for each iteration, and the number of iterations used before ending the algorithm were empirically tuned to optimize computational performance of the algorithm. If the variance of the distributions are very large, the neighboring stimuli become more spread out, which may lead to better exploration at the beginning of the process, but fails to converge as smoothly in the later iterations. Conversely, if the variance of the distributions are very small, the iterative perturbations from one solution to the next is much smaller allowing for smoother convergences, but in the early stages the algorithm takes a lot longer to converge.

In most cases, L^2^-norm was used as the optimization metric, for example to achieve an energy-optimal electrical stimulus waveform for causing a single action potential. For other applications described in this study we defined different optimization metrics (e.g., peak or total protein concentration for the genetic toggle switch), which can include multiple performance measures (e.g., combining stimulus L^2^-norm and a desynchronization index for optimizing a stimulus that maximally desynchronizes a group of coupled neurons).

### Validation using a stochastically-seeded gradient algorithm

Calculus of variations^50^ is a well-established analytical framework used to address optimization problems. We solved the variational problem directly using a stochastically-seeded gradient algorithm^27^, applied to three models (defined in Supplementary Text S1) representing different types of transitions: from quiescence to a spike in the Hodgkin-Huxley model; between sustained oscillation and quiescence in the FitzHugh-Nagumo model; and between two steady states in a genetic toggle switch model. The stochastically-seeded gradient algorithm generates a series of random stimuli, and using a gradient-based approach, calculates how the system will respond to minute changes in the stimulus. The algorithm iteratively uses these small gradients, or slopes, to converge towards the optimal solution. Stochastic seeding again enables the algorithm to explore a large solution space, revealing both the global optimal waveform as well as local optima^27^.

## Results

### Stochastic search using extrema features convergences quickly

**Figure 3:**
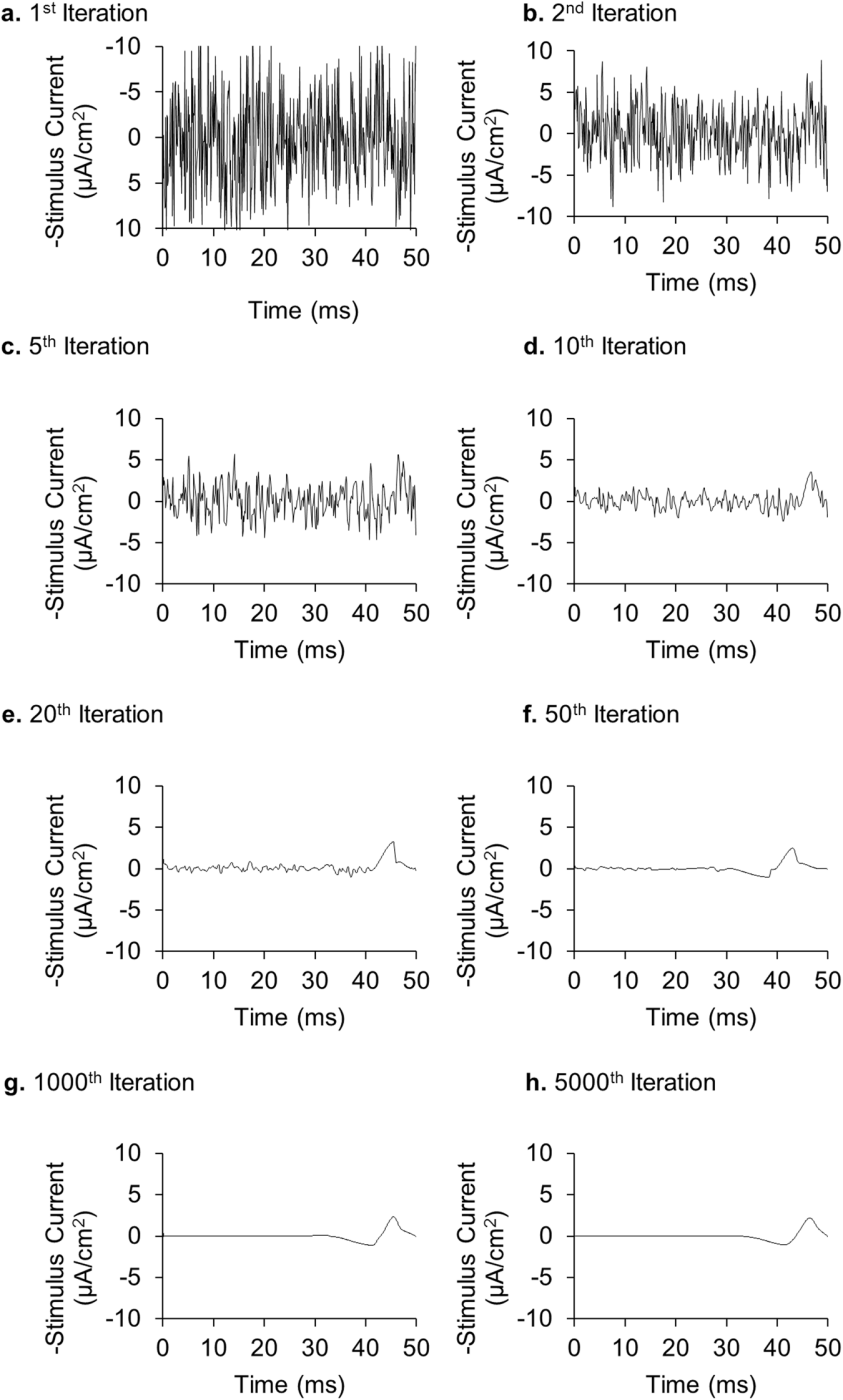
Convergence of the stimulus shape using an extrema feature stochastic search algorithm. We searched for the optimal 50-ms stimulus waveform necessary to trigger a single action potential. Waveforms are evaluated based on their L^2^-norm, or energy consumption. See Supplementary Text S1 for implementation details.

As described in the Methods section, this algorithm began with a stochastically generated stimulus. We first applied the algorithm to the Hodgkin-Huxley model to find the optimal stimulus waveform necessary to trigger a single action potential. Figure 3 shows the progression of this signal as the algorithm distorted the intervals between and amplitudes of the extrema. It is interesting to note that the algorithm converges very quickly in first few iterations and, within 10–20 iterations, the fundamental shape of the optimal solution appears. The next few thousand iterations further prune extraneous extrema and smooths the signal to converge towards the optimal solution.

**Figure 4:**
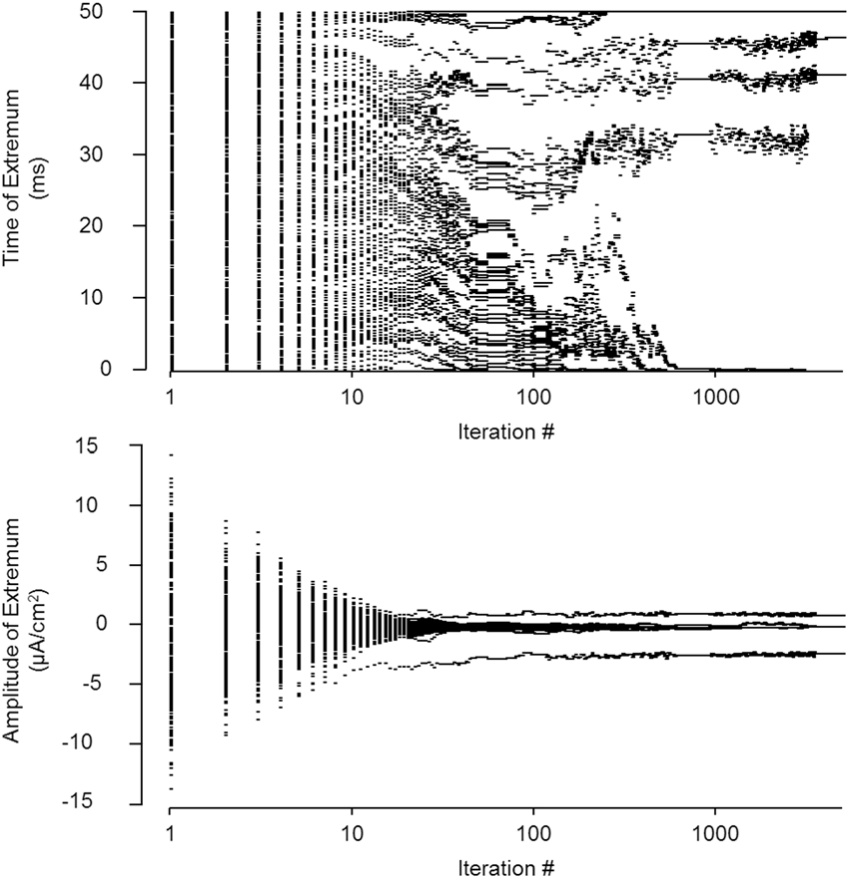
Tracking of the stimulus extrema. Every single point on both the top and the bottom panel represent a single extremum, plotted based either on when the extrema occurred in time (top) or in amplitude (bottom).

From a different perspective, one can observe the extrema, both in terms of their temporal locations and amplitudes as seen in Fig. 4. The algorithm removed extraneous extrema over the course of the algorithm.

### Using extrema improves search efficiency

**Figure 5:**
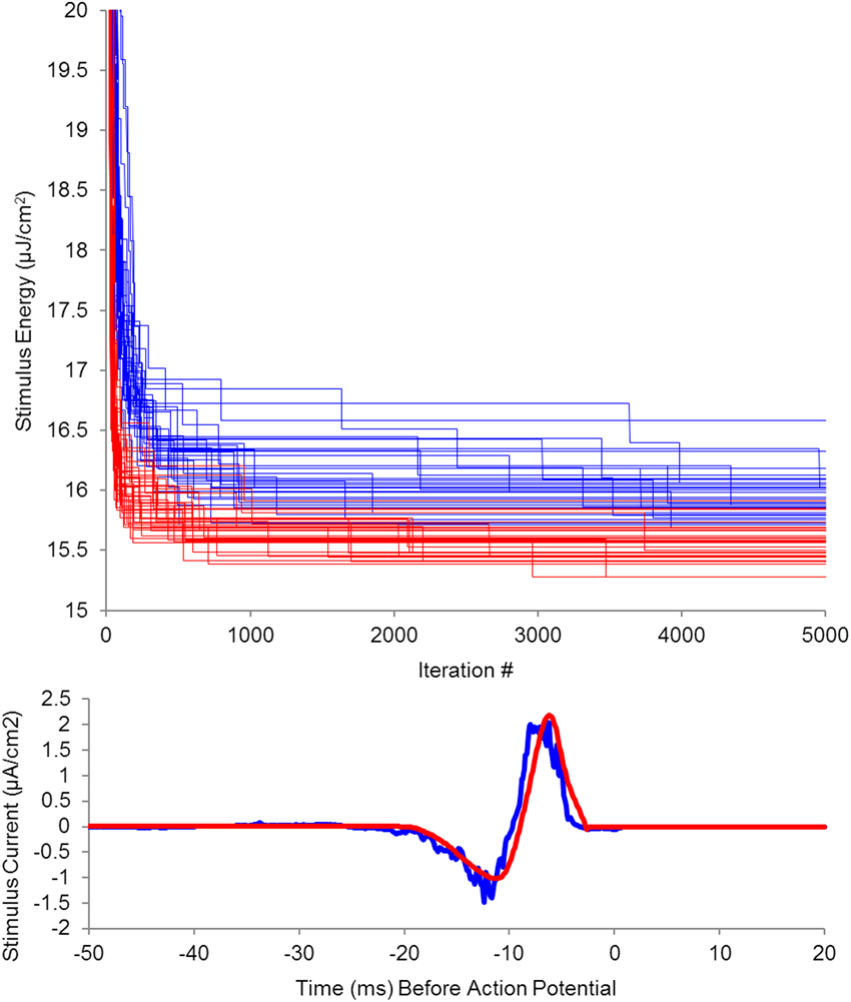
Comparison of an all-points search algorithm (blue) versus an extrema feature search algorithm (red). Top panel shows improvement in energy level (L^2^-norm) over the course of 500 iterations for 35 randomly seeded stimuli for each of the two algorithms. Bottom panel shows the stimulus for each algorithm having the least L2 norm after 500 iterations.

We reasoned that our focus on extrema would improve computational efficiency because it decreased the search space as we approached optimality. To evaluate this idea, we reconfigured our algorithm to use every single data point, i.e., stochastic perturbation of all-points instead of only the extrema points. As seen in Fig. 5A, the improvement of L^2^-norm across the 35 snippets using just the extrema converged towards an optimal solution faster than the improvement of L^2^-norm when perturbing all the points. The average number of iterations to drop below an L^2^-norm of 25 was 31.9 (S.D.: 5.5) using all-points versus 19.3 (S.D.: 5.2) using just extrema. To drop below an L^2^-norm of 20 took 51.6 iterations (S.D.: 15.6) using all-points versus 29.4 (S.D.: 7.8) using just extrema. To get below an L^2^-norm of 18 took 91.7 (S.D.: 34.5) iterations using all-points while it took only 36.8 iterations (S.D.: 9.4) using extrema only. Moreover, the average L^2^-norm when perturbing all points was 15.902 with a standard deviation of 0.171. The best result had an L^2^-norm of 15.683. Figure 5B shows that the best result in all-points perturbation retained a considerable level of noise as compared to the best result found when using just extrema.

Both results supported our hypothesis that using extrema features increases efficiency, while maintaining a high level of accuracy.

### Validation of stochastic search using extrema features

We applied this technique to three unique models to display the adaptability of this algorithm to a range of different systems. We studied two computational models used in neuroscience: the Hodgkin-Huxley model for triggering a single action potential, and the FitzHugh-Nagumo model for suppressing repetitive firing. We have also applied this algorithm to a model used in synthetic biology, the genetic toggle switch, for causing a switch from one steady state to another. The methods and models used are described in greater detail in Supplementary Text S1.

**Figure 6:**
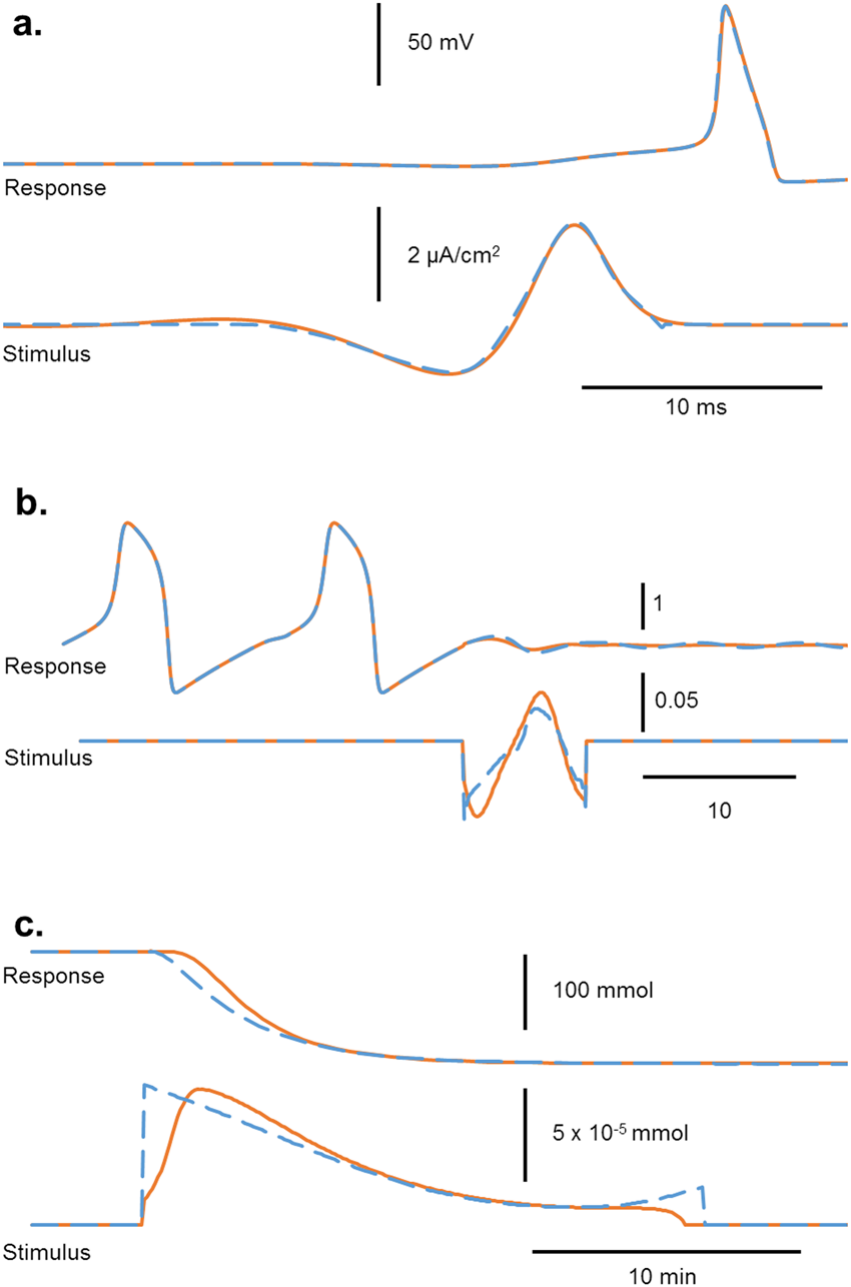
Comparison of optimal waveforms for eliciting a transition in three model. A single action potential in the Hodgkin-Huxley model (top), suppression of repetitive firing in the Fitzhugh-Nagumo model (middle), and induction of a switch in protein synthesis in the genetic toggle switch model (bottom), using the gradient algorithm (dashed blue) and our extrema search algorithm (solid orange).

We applied the algorithm to each of these models, and compared the resulting waveform obtained using the stochastically seeded gradient algorithm. Figure 6 shows the best solution from multiple runs with different starting seeds.

For the Hodgkin-Huxley model, this algorithm found solutions that had an average L^2^-norm of 15.6 (standard deviation of 0.149) compared to gradient algorithm at 15.4. For the FitzHugh-Nagumo model, results from focusing on extrema features had a mean of 7.2 × 10^−3^ (standard deviation: 1.8 × 10^−3^) compared to gradient algorithm’s 1.12 × 10^−2^. For the genetic toggle switch, the results had a mean of 2.97 × 10^−8^ (standard deviation: 2.25 × 10^−9^) compared to gradient algorithm’s 2.49 × 10^−8^.

In the FitzHugh-Nagumo and the Hodgkin-Huxley models, we noted that in certain instances, stochastic search did better than the gradient algorithm. Exploring the Hodgkin-Huxley model in greater depth, we noted that this technique only beat the gradient algorithm once in a set of 35 instances, having an L^2^-norm of 15.3. This is possible because the gradient algorithm is an approximation technique, utilizing first-order gradients to approach the optimal solution, and as such, depending on the topology of the system, it can have difficulty converging tightly to the optimal.

While this explained the discrepancy that occurred rarely with the Hodgkin-Huxley model, we were surprised that in the FitzHugh-Nagumo model, the stochastic search outperformed the gradient algorithm on a regular basis. As we investigated this further, we noticed that the extrema featured stochastic search found the optimal solution achieving our constraint, the suppression of repetitive firing, without necessarily having reached the mathematical stable point.

The FitzHugh-Nagumo model is interesting in that there is a stable limit cycle and a stable fixed point, with an unstable cycle in between the two states. The basin around the stable fixed point is very shallow, meaning that the system takes a long time within this basin to converge towards the fixed point. If the stimulus caused the system to cross the threshold from repetitive firing into quiescence, there will still be a period of oscillation depending on the state’s proximity to the stable fixed point. The gradient algorithm calculated the optimal solution for the system to reach very close to the fixed point by the end of the simulation, while our algorithm sought to simply cross the threshold from repetitive firing to quiescence in the same time frame. We demonstrated the energy needed to cross the threshold is much smaller than the energy necessary to take the system completely to the fixed point.

### Flexibility of stochastic search

**Figure 7:**
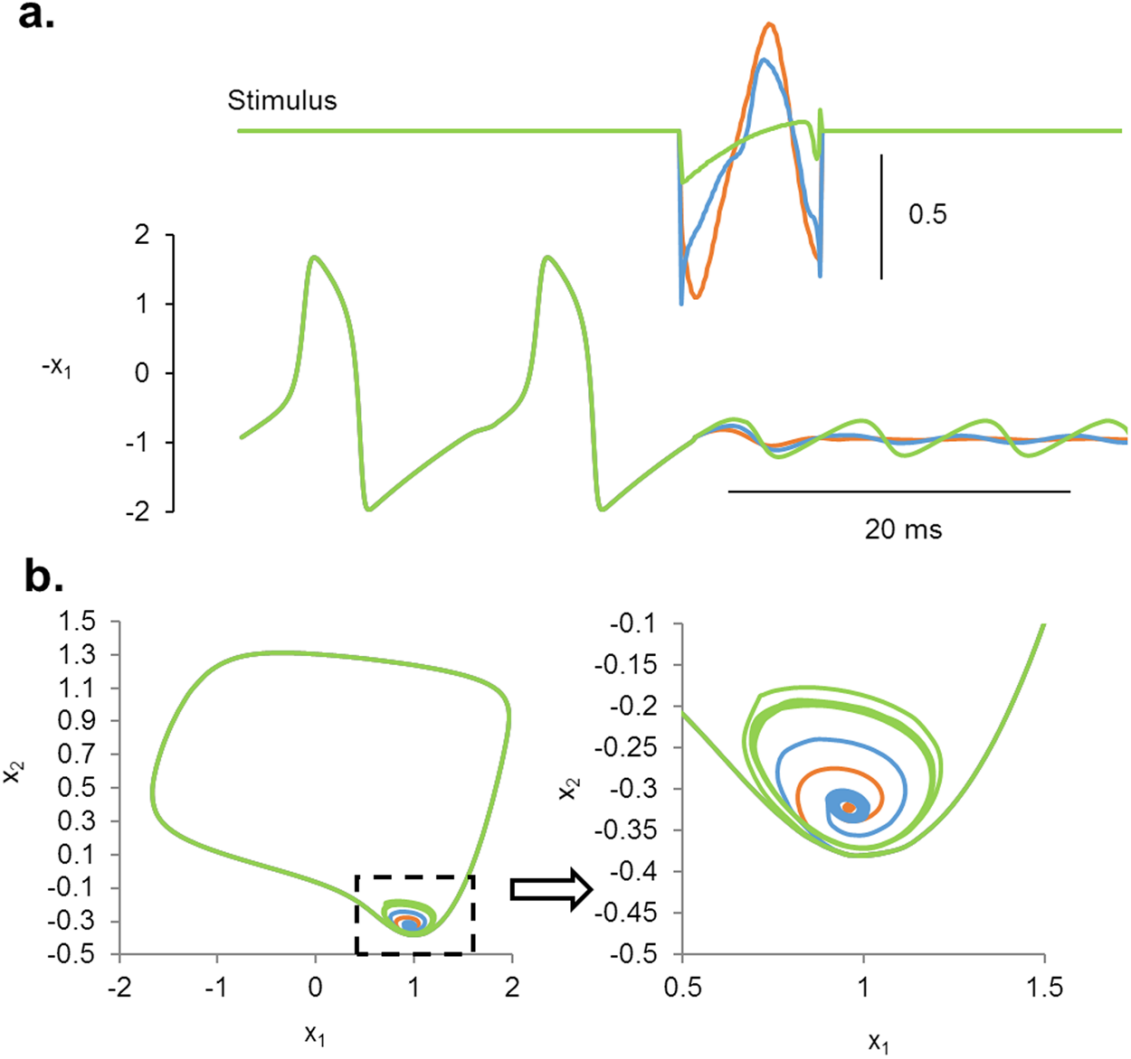
Searching for optimality using different levels of terminal condition constraints. Stricter constraints on the terminal condition in the FitzHugh-Nagumo model (blue) led to results that match more closely to the results of the gradient algorithm (gray) demonstrating a steadier quiescent state as compared to when looser constraints on the terminal condition were applied (orange).

Based on what we noticed in the FitzHugh-Nagumo model, we further investigated how changing the constraints affected the results. Could we tighten or loosen the constraints that define a successful suppression, and how would that affect the optimal solution? Fig. 7 shows the results of that experiment. We tightly suppressed the terminal oscillations in one experiment, with a resulting stimulus waveform that matched the gradient algorithm result much more closely. Similar, we also loosened the constraint such that only the bare minimum was required to suppress repetitive firing of action potentials. The result showed that a large energy difference could be achieved by this simple change in constraints. Using the gradient algorithm, we could not make these adjustments without defining the entire unstable limit cycle quantitatively and searched for optimal waveforms to every point along the unstable limit cycle. Here, our algorithm adjusted itself quickly to our changes in constraints.

**Figure 8:**
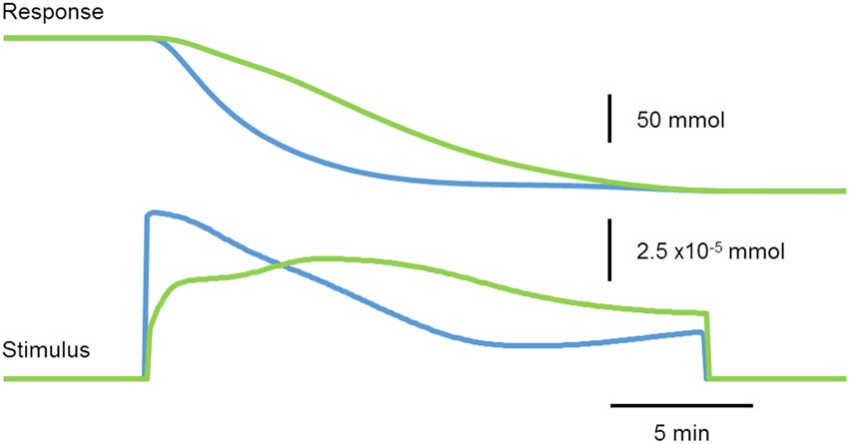
Optimal stimulus waveform is different depending on the performance metric defined. In the genetic toggle switch, minimizing peak dosage (red) revealed a different waveform than minimizing total concentration (blue).

Not only were we able to adjust our constraints, but we found that we could adjust our performance metrics to more real-world quantities in ways that are difficult to achieve mathematically. For instance, in a genetic toggle switch, the total concentration of molecular substrate used may not be as relevant as the peak dosage at any given point in time. It is difficult to write a mathematical formula defining peak dosage at any given point in time for the purposes of the gradient algorithm, but relatively straight forward with using our algorithm. Instead of using the L^2^-norm in our performance metric, we would use the maximal dosage amount in the stimulus as our performance metric. Figure 8 shows the differences between the two results using our algorithm: optimization for total concentration versus maximal dosage.

### Application of stochastic search using extrema features to complex systems – Desynchronization

**Figure 9:**
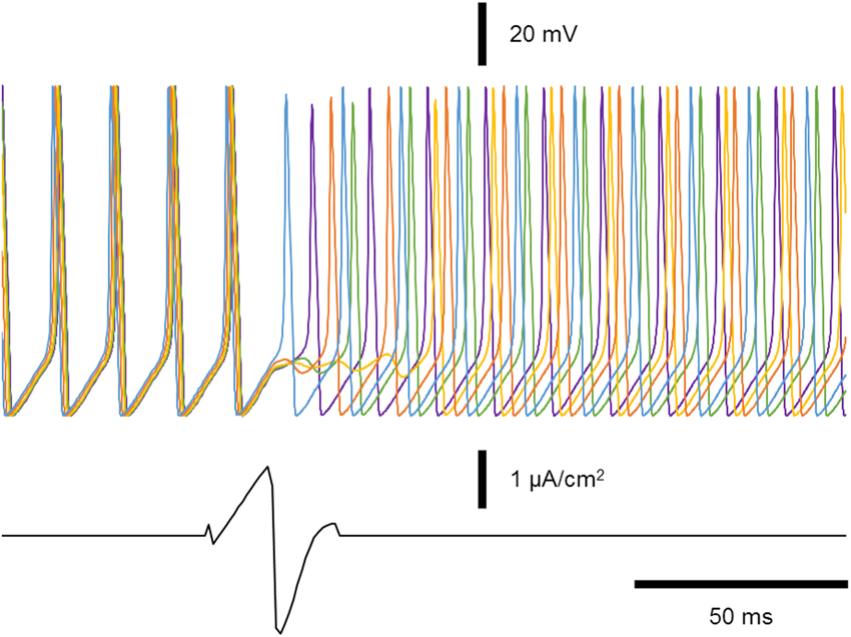
Desynchronization of coupled oscillator network. The stimulus shown here (bottom) is the optimal found after 1,000 iterations that maximized desynchronization seen in the coupled oscillators (top). The algorithm searched to minimize the mean voltage while also minimizing energy consumption. Positive axis for the stimulus reflect depolarization. While a numerical solution based on analytically-derived approaches for optimality for this model could not be calculated, we systematically tested a wide range of rectangular pulses with varying widths and amplitudes to find the optimal rectangular pulse. We found the optimal rectangular waveform had an L^2^-norm of 154.88 μJ/cm^2^ as compared to 11.80 μJ/cm^2^ for the optimal determined by our extrema-driven stochastic search algorithm.

We were able to also construct compounded requirements for the optimal stimulus waveform necessary to suppress synchronization of multiple repetitively firing neurons. This concept has been hypothesized to be important from a clinical perspective in the suppression of seizure activity^17,19,28,51^. Finding the optimal waveform for a complete ionic model of multiple coupled neurons is difficult; analytical approaches have attempted to solve simplified phase models^19,28,29^, but solutions to ionic models have not been reported. Our algorithm approached this easily by constructing a compound performance metric that simultaneously (1) minimized stimulus energy and (2) maximized neuronal desynchronization. In the example shown, the algorithm was programed to maximize neuronal desynchronization with a higher weight over the minimization of stimulus energy. Figure 9 shows the resulting optimal waveform pattern to desynchronize a group of five coupled Hodgkin-Huxley neurons. We used the mean field voltage as our marker for desynchronization, i.e., the lower the mean field voltage, the greater the neurons are desynchronized^19^. The model and methods can be found in Supplementary Text S1.

**Figure 10:**
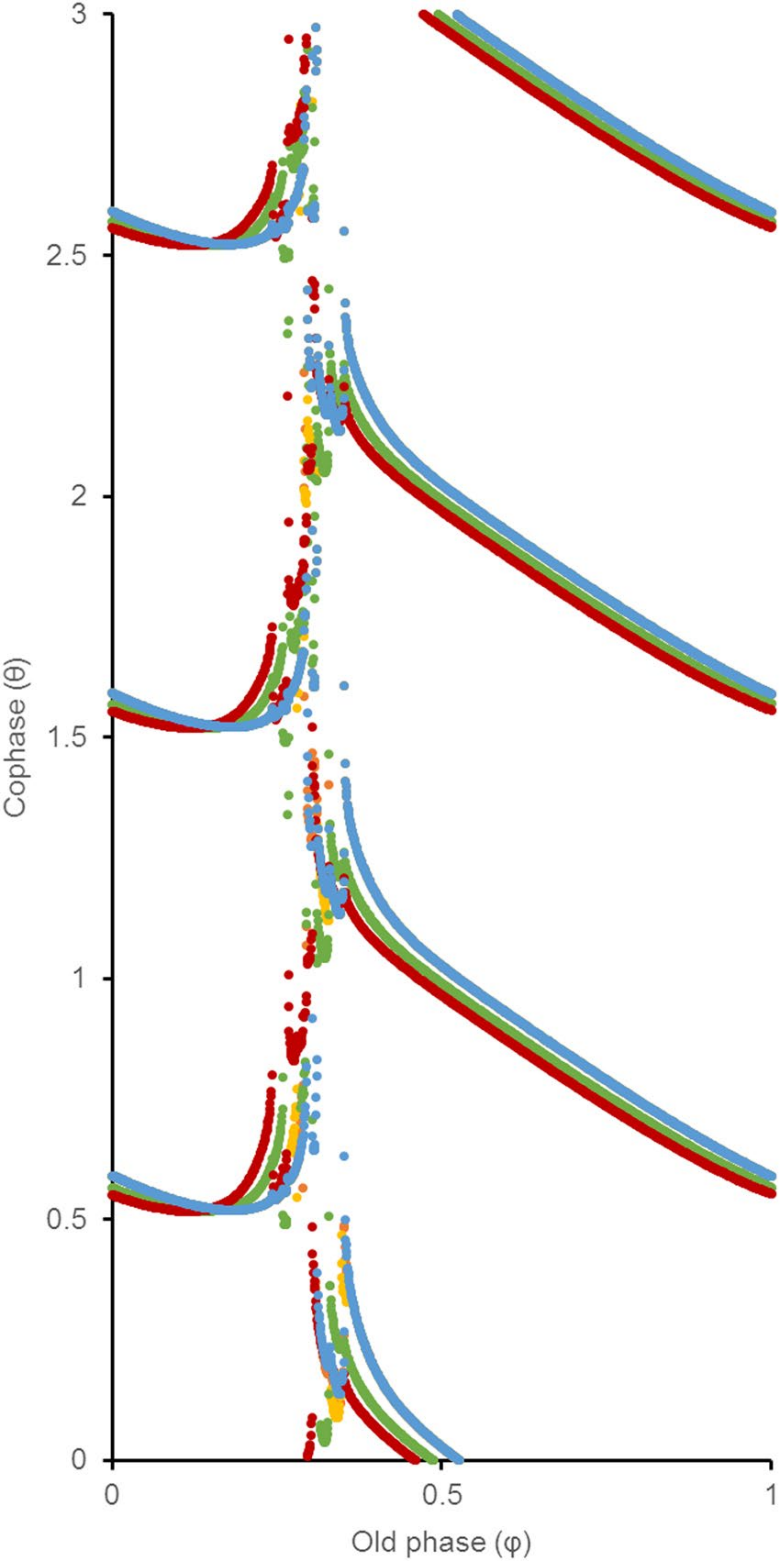
Phase resetting curves of the coupled oscillator network. Old phase (φ) is defined as the time, normalized to one cycle length, between the onset of the burst to the onset of the stimulus. Cophase (θ) is defined as the time, normalized to one cycle length, between the end of the stimulus and the next action potential for each of the neurons. We define the cycle length as the time between the onsets of two consecutive bursts immediately prior to stimulation. The data for each neuron is depicted with a different color.

We noted that the optimal stimulus exhibits a rapid reversal of polarity just before the expected burst of action potentials from the synchronized neurons. We believe that this is because the stimulus is being given close to a point of discontinuity on the phase resetting curve. Thus, as seen in Fig. 10, when the stimulus hits the system at this point, the tiny phase differences in the initial conditions are suddenly magnified, and the phases are dispersed.

Previous work has shown that the phase response of nonlinear oscillators to discrete perturbing stimuli can show similar phase dispersion in response to precisely timed stimuli. These studies use topological classification of phase-resetting curves to guide how stimulus strength and timing should be iterated towards a stimulus that induces maximum phase dispersion^52,53^. One limitation of the topological method is the presence of apparent discontinuities in phase resetting curves, which defies classification and complicates the search for successful stimuli^54–56^. Our results identify critically timed and shaped waveforms that are also optimized energetically, without the need for topological classification of resetting behavior.

### Optimal solutions can be phase dependent

It is important to note that in all of our optimization algorithms, the stimulus found is optimized specifically for the parameters by which we set up the problem. In the FitzHugh-Nagumo model, the optimization protocol that we examined is for a particular stimulus of a specified duration starting at a specific phase of the limit cycle. In the coupled oscillator model, the optimization protocol that we examined is for a particular stimulus of a specified duration starting at a particular set of initial conditions for synchronization. In both of these applications, the state transition occurs only within a narrow range of phases. When the stimulus is given at other phases, there is no state transition. Figure 10 illustrates this narrow window by plotting out the phase resetting curves of the five coupled oscillators in response to the stimulus. As we can see from this figure, the stimulus causes the 5 neurons to exhibit highly variable phases following stimulation (cophase, θ), only when the optimized stimulus is given within a narrow window of phases in the cycle (old phase, φ). When the stimulus is given at all other phases, the reset times (θ) of the 5 neurons do not exhibit dispersion of phases. Standard definitions of phase and cophase were used^52^.

### Optimal waveforms are non-rectangular

Using the method we described in our previous work^27^, we determined an optimal rectangular waveform pulse for each of the models we used. The optimal rectangular pulse energy for inducing an action potential in the Hodgkin-Huxley model is 49 µJ/cm^2^, compared to the optimal energy using our algorithm, 15.6 µJ/cm^2^. Suppressing repetitive firing in the FitzHugh-Nagumo model using an optimal rectangular pulse intensity (L^2^-norm) of 6.4 × 10^−2^ compared to waveform computed from our algorithm, 7.3 × 10^−3^. In the model of a network of coupled oscillators, the optimal rectangular pulse was 154.88 µJ/cm^2^, while the optimal we found was 11.80 µJ/cm^2^.

When examining the optimal stimulus waveform for the Hodgkin-Huxley model, we do note that there is a hyperpolarizing component followed by a depolarizing component. The ionic mechanisms of this particular shape are explored more fully by Clay *et al*.^57^. As previously suggested, these biphasic waveforms in ionic systems have resonant-like properties^58^.

Video illustrating the evolution of optimal waveforms and the system responses in the four models are available in Supplementary Videos S1–S4.

## Discussion

One of the challenges in understanding critical transitions is determining the ideal dynamical features of inputs necessary to induce them. This study sought to determine these ideal features by finding optimal stimulus waveforms necessary to induce state transitions by focusing on extrema features using a stochastic optimization approach. In three separate biological models, our approach developed solutions that closely match those found using traditional variational techniques.

There have been alternative model-independent techniques used in the past to find these optimal stimulus waveforms. Spike-triggered averaging was one such stochastic technique that computed the average input stimulus preceding each spike generated from broad-band stimulation^26,59^, though it was shown that the topology of the solution space had a large biasing effect away from optimality^40^. Stochastic search algorithms that leveraged evolutionary computation, like genetic algorithms, have also been applied to finding the optimal stimulus waveform^39^. This previous study used every point of the stimulus waveform as a distinct dimension, and thus required many iterations to converge towards a solution.

In order to reduce the dimensionality of the solution space, not of the model, we explored the idea of feature extraction. In the field of time-series analysis, feature extraction is a necessary step in solving problems like time series subsequence data classification^60–62^, signal pattern recognition^63–65^, and anomaly detection^66,67^ as often the data set is so large that huge amounts of computational power would be necessary to compare and analyze every data point in the set. While many studies use features like Fourier coefficients or eigenvectors^64,68^, there has been increasing interest in the use of extrema because of its ease of capture, relationship to variation in the data, and resilience in the face of disruptions^49,65,69^. To our knowledge, ours is the first study on the use of extrema applied to search for optimal stimulus waveforms.

As we have shown, extracting the extrema and using them as the key feature points of the stochastic search algorithm has greatly improved the results of the algorithm. From this foundation, we believe that the algorithm can be further developed for greater improvement: initial noise reduction, statistical learning, and further feature analysis and extraction. We discuss in Supplementary Text S2 some of the preliminary work that we did in the reducing the noise of the starting seed, our findings and areas of further exploration. With regards to statistical learning, the algorithm as it currently stands utilizes a memoryless Gaussian distribution in order to choose the next stimulus to try. If we can incorporate memory into the algorithm, we may be more efficient with our search protocols such that the algorithm explores unknown solutions more frequently than repeating waveforms similar to ones that it has tested previously. Finally, in our existing protocol, we utilize only the first order extrema points (e.g. local minima and maxima) causing us to lose some information. We have not characterized or quantified the information loss by using only the first-order extrema, but future studies may help us gain a better appreciation of how much information is lost, whether greater orders of extrema (such as the inflection points between extrema) are necessary to retain as much characterization of the waveform while still maintaining a certain level of computational efficiency. Furthermore, by gaining a better understanding of the robustness of each data point, we may gain more insight with regards to other potential landmarks to use.

In our exploration into computational biology, we have found many more biological models that can be studied, from cardiology (examining cardioversion or defibrillation from arrhythmias^70–72^) to respiratory (stimulating respiratory rhythms in patients with apnea)^7^, pharmaceutical sciences (studying optimal dosing patterns for drug treatments^73,74^). Furthermore, it may be interesting to model not just the behavior of the biological system in response to electrical stimuli, but also the electrical and physical components that interface with it (e.g. electrodes, circuits, chips). As some researchers have noted, while we may be able to cut down on the energy consumption of the signal itself, the energetic requirements of the hardware may overwhelm any energy savings on the stimulus waveform itself^34^. Searching for optimality in the entire system from electrode to biology may yield further insights into how we can improve the biological response to exogenous stimuli.

The use of extrema features in stochastic search could also potentially be used to not just search for transitions between multiple distinct states, but also perhaps to shift systems along a continuum, like in the phase resetting of clocks. What is the optimal light therapy necessary to minimize jet lag or adverse effects of shift work ^53,75,76^? While some are tackling this from an analytical and quantitative perspective using models, perhaps different insights may be gained from approaching the problem with a model-independent perspective.

## Electronic supplementary material

Supplementary Information

Evolution of optimal stimulus in triggering an action potential in the Hodgkin-Huxley model using the extrema distortion algorithm

Evolution of optimal stimulus in suppressing repetitive firing in the FitzHugh-Nagumo model using the extrema distortion algorithm

Evolution of optimal stimulus in toggling a genetic toggle switch using the extrema distortion algorithm

Evolution of optimal stimulus in desynchronizing coupled network oscillators using the extrema distortion algorithm
