# Author Correction: Evolution of extrema features reveals optimal stimuli for biological state transitions

**DOI:** 10.1038/s41598-018-29098-y

**Published:** 2018-07-18

**Authors:** Joshua Chang, David Paydarfar

**Affiliations:** 1 Department of Neurology, University of Massachusetts Medical School, Worcester, Massachusetts 01604 USA; 2 Department of Neurology, Dell Medical School, The University of Texas at Austin, Austin, Texas 78701 USA; 3 The Institute for Computational Engineering and Sciences, The University of Texas at Austin, Austin, Texas 78701 USA

Correction to: *Scientific Reports* 10.1038/s41598-018-21761-8, published online 21 Febuary 2018

In this Article, the legend of Figure 6 is incorrect:

“Comparison of optimal waveforms for eliciting a transition in three model. A single action potential in the Hodgkin-Huxley model (top), suppression of repetitive firing in the Fitzhugh-Nagumo model (middle), and induction of a switch in protein synthesis in the genetic toggle switch model (bottom), using the gradient algorithm (dashed blue) and our extrema search algorithm (solid orange).”

should read:

“Comparison of optimal waveforms for eliciting a transition in three model. A single action potential in the Hodgkin-Huxley model (top), suppression of repetitive firing in the Fitzhugh-Nagumo model (middle), and induction of a switch in protein synthesis in the genetic toggle switch model (bottom), using the gradient algorithm (solid orange) and our extrema search algorithm (dashed blue).”

