# Supplementary material for: Evolution of extrema features reveals optimal stimuli for biological state transitions
Source: Sci Rep. 2018 Feb 21;8:3403. doi: 10.1038/s41598-018-21761-8 (PMC5821862; doi:10.1038/s41598-018-21761-8)
Supplement: Supplementary file 1 — Supplementary Information [file 41598_2018_21761_MOESM1_ESM.pdf]

Evolution of extrema features reveals optimal stimuli for

biological state transitions

Supplement S1: Descriptions of models

Joshua Chang[1,2], David Paydarfar[2,3]

[1] Department of Neurology, University of Massachusetts Medical School, 55 N Lake Ave, Worcester, Massachusetts, 01604. [2] Department of Neurology, Dell Medical School, and [3] The Institute for Computational Engineering and Sciences, The University of Texas at Austin, 1501 Red River St, Austin, Texas, 78701.

Correspondence and requests for materials should be addressed to J.C. (email: joshua.chang@austin.utexas.edu) or D.P. (email: david.paydarfar@austin.utexas.edu).

Hodgkin-Huxley model

The HH differential equations[43] describe a four-dimensional system that captures the ionic mechanisms underlying the firing of an action potential in a single neuron. We define our optimization problem as follows: finding the energy-optimal (defined by L^2-norm) stimulus that causes the neuron to fire an action potential within a specified time period.

In this case, we used the standard HH equations without a persistent injected current. The HH model equations are:

$$C\dot{V} = -120m^3h(V-115) - 36n^4(V+12) - 0.3(V-10.613) - I_l - u \quad (1)$$
$$\dot{m} = -m\big(\alpha_m(V) + \beta_m(V)\big) + \alpha_m(V) \quad (2)$$
$$\dot{n} = -n\big(\alpha_n(V) + \beta_n(V)\big) + \alpha_n(V) \quad (3)$$
$$\dot{h} = -h\big(\alpha_h(V) + \beta_h(V)\big) + \alpha_h(V) \quad (4)$$

where,

$$\alpha_m(V) = \frac{0.1\phi(25-V)}{e^{0.1(25-V)}-1}, \beta_m(V) = 4\phi e^{-V/80} \quad (5,6)$$
$$\alpha_n(V) = \frac{0.01\phi(10-V)}{e^{0.1(10-V)}-1}, \beta_n(V) = 0.125\phi e^{-V/80} \quad (7,8)$$
$$\alpha_h(V) = 0.07\phi e^{-V/20}, \beta_m(V) = \frac{\phi}{e^{0.1(30-V)}+1} \quad (9,10)$$

V is the membrane voltage, m, n, and h represent dimensionless quantities associated with sodium channel activation, potassium channel activation, and sodium channel inactivation respectively, I_l is the persistent leak current and u represents the exogenous stimulus into the system.

The Hodgkin-Huxley model that we have used has a resonant frequency of 60 Hz, and the eigenvalues at the stationary state are -4.6742, -0.1207, -0.2031 + 0.3831i, and -0.2031-0.3831i.

FitzHugh-Nagumo model

The FitzHugh-Nagumo model equations[44,45] describes excitable dynamics abstractly in two-dimensions and has been used widely to model diverse biological systems[77-80].

$$\dot{x_1} = c\left(x_2 + x_1 - \frac{{x_1}^3}{3} - r\right) + u \tag{11}$$

$$\dot{x_2} = -\frac{1}{c}(x_1 - a + bx_2) \tag{12}$$

The model is unitless, but when modeling neuronal excitability, x_1 is analogous to Hodgkin-Huxley's V and m, while x_2 is analogous to Hodgkin-Huxley's h and n state variables. The variable u represents the current stimulation, which can be in the form of an endogenous persistent current or an exogenous input stimulus. Using the parameters[81] $a = 0.7$, $b = 0.8$, $c = 3.0$ and $r = 0.342$, the system gravitates, when there is no stimulation, towards one of two states: quiescence (stable fixed point) or repetitive firing (stable oscillatory limit cycle). The minimum value of x_1 is the equivalent of the peak of an action potential. In the present study, we are looking to minimize the stimulus energy needed to toggle the system from the oscillatory state to the quiescent state. We have previously determine the optimal shape and phase angle for the start of an 8-ms stimulus waveform[27]. Thus, using the same parameters, we chose to apply the stimulus under the same starting conditions for the system ($x_1 = 0.9302$, $x_2 = -0.3760$).

As a note, the period of the unstable limit cycle in this model is around 7.27, while the period of the stable limit cycle is around 12.9.

Genetic toggle switch model

A variety of gene-regulatory networks are bistable and transient stimuli - molecular or thermal – can trigger the network to flip from one state to the other. A major advance in synthetic biology has been construction of a synthetic genetic toggle switch[46], composed of two repressors and two constitutive promoters. The opposing promoter transcribes each repressor. This model consists of two state variables, u and v, representing the two repressor concentrations. The equations governing this system are:

$$\frac{du}{dt} = \frac{\alpha_1}{1 + v^{\beta}} - u \tag{13}$$

$$\frac{dv}{dt} = \frac{\alpha_2}{1 + u^{\gamma}} - v \tag{14}$$

where α_1 and α_2 are the rate of synthesis of repressor 1 and repressor 2 respectively, β is the cooperativity of repression of promoter 2 and γ is the cooperativity of repression of promoter 1. The essential components of the synthetic GTS are a single plasmid that used the Lac repressor (lacI) in conjunction with the Ptrc-2 promoter as repressor 1 and a temperature-sensitive λ repressor (cIts), in conjunction with a P_{LS}1con promoter as repressor 2. Because of this setup, the parameters of the model are $\alpha_1 = 156.25$, $\alpha_2 = 15.6$, $\beta = 2.5$, and $\gamma = 1$.

In this toggle switch, only one of the concentrations is elevated at any given time. A pulse of isopropyl β-D-thiogalactopyranoside (IPTG) is applied to switch the system so that the lacI concentration is raised. A thermal pulse is used to switch the system back. The model includes IPTG as the exogenous stimulus to the system.

$$\frac{dv}{dt} = \frac{\alpha_2}{1 + \left(\frac{u}{\left(1 + \frac{[IPTG]}{K}\right)^{\eta}} \right)^{\gamma}} - v \tag{15}$$

where K is the dissociation constant of IPTG from LacR and η is the cooperativity of IPTG binding. K is set at 2.9618 x 10^{-5}, and $\eta = 2.0015$. Using the model, we can calculate the stable points by setting $\frac{du}{dt}$ and $\frac{dv}{dt}$ to zero. We learn that there are two stable fixed points: (0.3319, 11.7080) and (155.5143, 0.0995). An unstable fixed point was calculated at (1.3144, 6.7342).

From the literature, we find that a 20-minute pulse of IPTG is used to constitutively turn on lacI protein production. Our goal was to find the optimal 20-minute pulse shape of IPTG that would turn off the lacI protein production at the end of 20 minutes. One of the twists regarding the genetic toggle switch model is that the stimuli need to be positive-only because the stimulus represents the concentration of IPTG. A negative concentration is impossible. Thus, when finding our starting snippets, we tested 20-minute discrete non-negative intensity snippets. We generated white noise using a uniform distribution random number generate with an amplitude of 0.0002 and a resolution of 0.1 minutes, capturing 29 snippets out of 50 snippets. In the distortion phase, instead of adding a randomly generated number to the peak, we multiplied the peak by a randomly generated number with a Gaussian distribution with mean of 1 and standard deviation of 0.1. This way, the values would stay positive. For the genetic toggle switch model, we multiplied the time intervals by a randomly generated number with a Gaussian distribution with mean of 1 and standard deviation of 0.25. We ran the algorithm for 2,000 iterations.

Coupled oscillators network

From a network perspective, there is a hypothesis that one of the mechanisms underlying epileptic seizures is a synchronization of various regions of the brain[82-85]. Synchronization has been observed in rat hippocampal slices perfused with high potassium saline solutions[86]. One of the alternative electrical therapies for patients has been vagal nerve stimulation (VNS). While its exact mechanism is still unknown, it is hypothesized that VNS works by desynchronizing neuronal activity, an idea supported by the EEG response to VNS[51].

We have modeled a network of five coupled Hodgkin-Huxley neurons to evaluate the efficacy of this algorithm to promote desynchronization. We chose five for computational purposes, but the model can be scaled up relatively easily. To model them as oscillators, we have increased the persistent current to 12 μA/cm^2 such that each neuron is a monostable oscillator exhibiting repetitive firing[87]. We coupled every neuron with every other neuron by using a constant coupling factor and the voltage differences between the neurons:

$$\alpha \sum_{j=1}^{5} \left(V_i - V_j\right) \tag{16}$$

where i represents the neuron in question, j represents all neurons and α is the coupling constant. The new model of equations replaces Eq. (1) with:

$$\begin{aligned} C\dot{V}_i = -120{m_i}^3 h_i(V_i - 115) - 36{n_i}^4(V_i + 12) - 0.3(V_i - 10.613) - 12 \\ - \alpha \sum_{j=1}^{5} \left(V_i - V_j\right) - u \end{aligned} \tag{17}$$

where V_i, m_i, n_i, and $h_i$are the state variables of the ith neuron. All the other equations still applied. We chose a coupling coefficient of 0.01. When the five oscillators are synchronized the peak mean field potential is approximately 94 mV. When the phases of the five oscillators are evenly dispersed across the cycle (i.e., fully desynchronized), the peak mean field potential drops to a minimum around 20 mV.

Numerical simulations show that using a coupling coefficient of 0.01 the system converges towards a synchronized state, regardless of the initial conditions. We found this to be the case for 100 simulations using random initial conditions, including cases in which the initial phases of the 5 neurons are dispersed evenly across the cycle. Figure S1.1 shows examples of convergence for 20 randomly chosen initial conditions

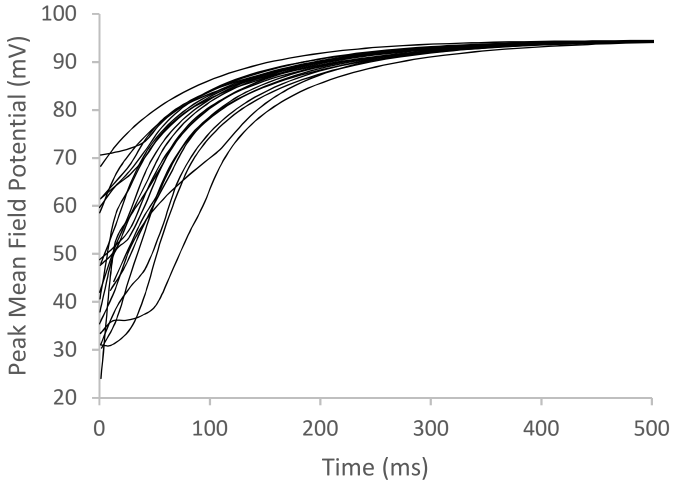

Figure S1.1. *Convergence of the system at various initial conditions to synchronization.*

We introduced the stimulus before the system converged completely to synchronization. The initial conditions were set such that the mean field potential peak was approximately 72.5 mV and the spread of the phases of the oscillators were within 10% of the cycle length of the mean field potential. A 31-ms stimulus duration (two cycle lengths) was given with the goal to maximally suppress the mean field potential, while minimizing the stimulus waveform energy. The performance metric was set such that suppression of the mean field potential was weighed more heavily compared to the minimizing of stimulus waveform energy. In our experiments, the system would begin to converge towards synchrony again shortly after the stimulus was given as illustrated in Figure S1.1, and so we measured only the peak mean field potential within 100 ms of the end of the stimulus.

Devices giving electrical charge to the body attempt to do so in such a way that the net electrical charge injected is equal to zero. Damage to the tissue due to charge imbalanced stimulation has been proposed along different mechanisms including the movement of charged components of the cell out of their physiological locations (e.g. proteins), and the formation of toxic electrochemical reaction products due to charge deposition in the cells[88,89]. As such, we added a new constraint to our algorithm such that after every distortion, we subtract the mean of the stimulus from the stimulus. This forces each distortion to produce only zero-mean stimulus waveforms. Thus, we could restrict the search space for all the experiments to return only charge-neutral solutions. We ran each experiment twenty times with different starting seeds each time.

Evolution of extrema features reveals optimal stimuli for biological state transitions

Supplement S2: Pre-filtering our starting seed

Joshua Chang[1,2], David Paydarfar[2,3]

[1] Department of Neurology, University of Massachusetts Medical School, 55 N Lake Ave, Worcester, Massachusetts, 01604. [2] Department of Neurology, Dell Medical School, and [3] The Institute for Computational Engineering and Sciences, The University of Texas at Austin, 1501 Red River St, Austin, Texas, 78701.

Correspondence and requests for materials should be addressed to J.C. (email: joshua.chang@austin.utexas.edu) or D.P. (email: david.paydarfar@austin.utexas.edu).

During this work, we speculated that some initial pre-processing filter of the noise in the seed would be useful to increase the efficiency of the algorithm. As such, we explored this idea by using empirical mode decomposition[90,91] to break down the successful white noise snippets into what we termed "distilled" snippets. These distilled snippets were then processed through the extrema distortion algorithm, allowing us to evaluate the benefits of the noise filter.

The idea behind this concept is that pure white noise snippets contain many extrema. If we could reduce the number of extrema down to just the right number of extrema, each iteration of the distortion algorithm would require fewer computations, and thus over all the algorithm would be more efficient.

To distill the snippets down, we considered using Fourier analysis, wavelet analysis and empirical mode decomposition to break down the white noise snippets to determine the fundamental signal underlying the snippet that is successful in causing the state transition. We decided to use the empirical mode decomposition. Because we were working with short discrete stimuli, we did not want to use Fourier analysis so as not to deal with edge effects. Furthermore, we chose not to use wavelet analysis because we understood that the choice of wavelets might affect the results.

Thus, we chose to use empirical mode decomposition to separate out the white noise snippets into a set of intrinsic mode functions (IMFs). We then tested every combination of consecutive IMFs, scaling each combination until it just barely caused a state transition. From these results, we chose the combination of consecutive IMFs that used the least amount of energy.

The distillation process can reduce many of the white noise snippets into very energy efficient snippets that successfully cause a state transition with a mean L^2-norm of 29.206 and standard

deviation of 8.256. We then give each one of these distilled snippets to the extrema distortion algorithm. The resulting distilled and distorted snippets have a mean L^2-norm of 16.695 and standard deviation of 1.3194.

Fig S2.1 shows that compared to just during extrema feature stochastic search alone with no filtering (mean = 15.550, standard deviation = 0.149), running EMD first caused a wider spread in results from this algorithm. Furthermore, the best result from doing the pre-processing had an L^2-norm of 15.438, which was comparable to the 15.275 from the stochastic search alone. Fig S2.2 shows the best result from the stochastic search with and without pre-processing filter.

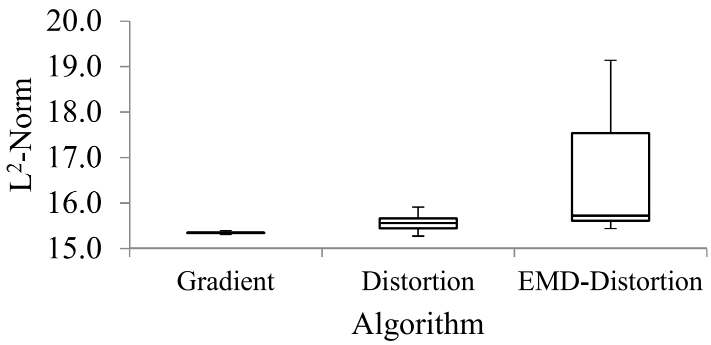

Figure S2.1. *Comparison of the ranges of L^2-norm results between the gradient algorithm, the stochastic search using distortion of extrema features, and the empirical mode decomposition combined with the extrema distortion algorithm.*

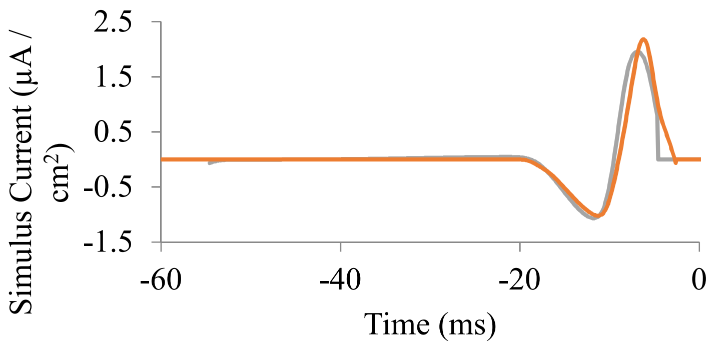

Figure S2.2. *Comparison of the best result from stochastic search using extrema feature algorithm (red) with the best result from the algorithm combined with an initial empirical mode decomposition denoising. The stimulus is aligned such that the action potential occurs at 0 ms.*

It is interesting to note that there is no correlation between how well a snippet did after the distillation phase as compared to the perturbation phase. Furthermore, the distillation process produced solutions that were worse than those generated from using only distortion. Our conclusion is that it is possible for any sort of pre-processing work to over simplify the white

noise snippets, biasing the signal away from optimality. Future research and algorithm development may show how better to reduce the amount of noise to start from the white noise snippet, while not overlying biasing the solution.

References for Supplements S1 and S2

77. Perthame, B. *Transport Equations in Biology.* (Springer Science & Business Media, 2006).
78. Aliev, R. R. & Panfilov, A. V. A simple two-variable model of cardiac excitation. *Chaos, Solitons & Fractals* **7,** 293–301 (1996).
79. Cantrell, S., Cosner, C. & Ruan, S. *Spatial Ecology.* (CRC Press, 2009).
80. Dokos, S., Cloherty, S. L. & Lovell, N. H. Computational model of atrial electrical activation and propagation. *Annu. Int. Conf. IEEE Eng. Med. Biol. - Proc.* 908–911 (2007).
81. Paydarfar, D. & Buerkel, D. M. Dysrhythmias of the respiratory oscillator. *Chaos* **5,** 18–29 (1995).
82. Jirsa, V. K., Stacey, W. C., Quilichini, P. P., Ivanov, A. I. & Bernard, C. On the nature of seizure dynamics. *Brain* 2210–2230 (2014).
83. Truccolo, W. et al. Neuronal ensemble synchrony during human focal seizures. *J. Neurosci.* **34,** 9927–44 (2014).
84. Jiruska, P. et al. Synchronization and desynchronization in epilepsy: controversies and hypotheses. *J. Physiol.* **591,** 787–97 (2013).
85. Jefferys, J. G. & Haas, H. L. Synchronized bursting of CA1 hippocampal pyramidal cells in the absence of synaptic transmission. *Nature* **300,** 448–450 (1982).
86. Jensen, M. S. & Yaari, Y. Role of intrinsic burst firing, potassium accumulation, and electrical coupling in the elevated potassium model of hippocampal epilepsy. *J. Neurophysiol.* **77,** 1224–1233 (1997).
87. Rinzel, J. On repetitive activity in nerve. *Fed. Proc.* **37,** 2793–2802 (1978).
88. Lilly, J. C., Hughes, J. R., Alvord, E. C. & Galkin, T. W. Brief, Noninjurious Electric Waveform for Stimulation of the Brain. *Science.* **121,** 468–469 (1955).
89. Merrill, D. R., Bikson, M. & Jefferys, J. G. R. Electrical stimulation of excitable tissue: Design of efficacious and safe protocols. *J. Neurosci. Methods* **141,** 171–198 (2005).
90. Huang, N. et al. The empirical mode decomposition and the Hilbert spectrum for nonlinear and non-stationary time series analysis. *Proc. R. Soc. London A Math. Phys. Eng. Sci.* **454,** 903–995 (1998).
91. Peng, C. K., Costa, M. & Goldberger, A. L. Adaptive Data Analysis of Complex Fluctuations in Physiologic Time Series. *Adv Adapt Data Anal* **1,** 61–70 (2009).
